# Simultaneous measurement of two biological signals using a multi-layered polyvinylidene fluoride sensor

**DOI:** 10.1038/s41598-022-05622-z

**Published:** 2022-01-27

**Authors:** Long-Nguyen Manh, Jinghua Li, Hyunkyu Kweon, Younghun Chae

**Affiliations:** 1 Department of Mechanical System Engineering, Kumoh National Institute of Technology, Gumi-si, 39177 Korea; 2 Department of Mechanical Engineering, Kyungpook National University, Daegu, 41566 Korea

**Keywords:** Biological techniques, Nanoscience and technology

## Abstract

Cardiovascular and deep breathing diseases can be detected by measuring human signals such as heart rate, respiration, and blood pressure, which are important physiological parameters for accessing the state of the body. However, conventionally, heart and respiration rates are monitored using different sensors, which is cumbersome and can further increase the psychological burden on patients. To address these issues, this report proposes a sensor consisting of two stacked elements that can simultaneously measure heart and respiration rates. The two signals received can be expressed separately as heart and respiration rates after signal processing. The two stacked elements are composed of polyvinylidene fluoride thin film bonded to a polydimethylsiloxane substrate. One element (element 1) measures movement related to the heart, and the other (element 2) measures movement related to breathing. Elements 1 and 2 were experimentally observed to have sensitivities of 0.163 V/N and 0.209 V/N, respectively. In addition, the proposed system was compared with a commercial digital heart rate and respiration rate measurement instrument and was verified to be effective for simultaneous measurement of human vital signals with multiple sensors. In addition, the proposed system is flexible, lightweight, and inexpensive, making it convenient and economical.

## Introduction

## Background

According to the World Health Organization (WHO), many health fern systems are being researched and developed because mental stress increases stress on the cardiovascular and cardiopulmonary systems. These diseases can be diagnosed by measuring signals indicating the vital signs, such as heart rate, respiration rate, and blood pressure. However, it is important that these vital signals be measured easily and quickly in homes and clinical settings.

In hospitals, chest movements, nose and mouth breathing, and electrocardiography (ECG) are used as non-invasive methods to measure heart rate and breathing^1,2^. This may help doctors diagnose, but there are certain restrictions. First, one device can measure only one signal, that is, either the heart rate or breathing. Second, the user’s proficiency is required to increase the reliability of the measurement signal. Third, the equipment is complex and expensive and can only be used in clinical settings.

It is necessary to develop a device that can simply measure two signals to predict the risk of acute heart attack and sleep apnea in patients through changes in heart rate and respiration rate. Hence, this study proposes a method for simultaneously measuring the heart rate and respiration rate.

Sensors using polyvinylidene fluoride (PVDF), a popular piezoelectric material, have been proven to be effective in detecting vital signals. Many studies have proven that PVDF is very effective in measuring the biological signals of the human body^3–6^. Using PVDF, an equipment that can be used at home was developed. A developmental product consisting of a PVDF cable sensor and a PVDF sensor array can measure the heart rate and respiration rate of a sleeping person^7^. In addition, wearable belt-type PVDF and fiber materials measure the heart rate and respiration rate, respectively^8^.

Some of these signals obtained using PVDF are noisy owing to body movements^9^. Heart rate and respiration signals have low frequencies with a frequency range of 1 to 2 Hz (60 to 120 times/min) for the heart rate, and the breathing occurs at a frequency of 0.2 to 0.34 Hz (12–20 times/min) and a pressure of several KPa^10^.

This study proposes a new method. i.e., a stacked PVDF sensor. This sensor can measure the heart rate and respiration rate simultaneously and is made of two elements by stacking four layers of polydimethyl siloxane (PDMS) and PVDF thin film alternately. Here, one element (element 2) measures the respiration rate, and the other element (element 1) measures the heart rate signal. It uses OP-amps, resistors, and capacitors to measure the signal.

## Physiological theory

### Heart rate

The heart rate was determined using ECG. An ECG tracing of a single heartbeat consists of a P wave, QRS wave, and T-wave as shown in Supplementary Figure S1. The P wave corresponds to arterial depolarization, QRS wave is the QRS complex for ventricular depolarization, and the T-wave corresponds to ventricular repolarization.

### Respiration

The breathing process consists of inhalation and exhalation. When a person inhales, their lungs expand and then contract on exhalation. In general, the respiration rate is 12–20 breaths/min (frequency: 0.2–0.34 Hz). Supplementary Figure S2 shows the respiratory signals measured using a PVDF sensor.

## Sensor design

### Measurement method

There are several ways to monitor heart rate and breathing. This study measures heart rate and respiration simultaneously by employing one sensor. The working mechanism of the sensor is based on periodic vibration and vibrational deformation of the human chest during the blood pumping action of the heart and breathing. Heart rate and breathing not only affect the movement of the chest but also the body movement to some extent. Therefore, these signals were measured using complex measurements and filtering. Both heart rate and respiration rate were measured, as shown in Supplementary Figure S2, using one PVDF thin film, but it is difficult to separate them.

**Figure 1:**
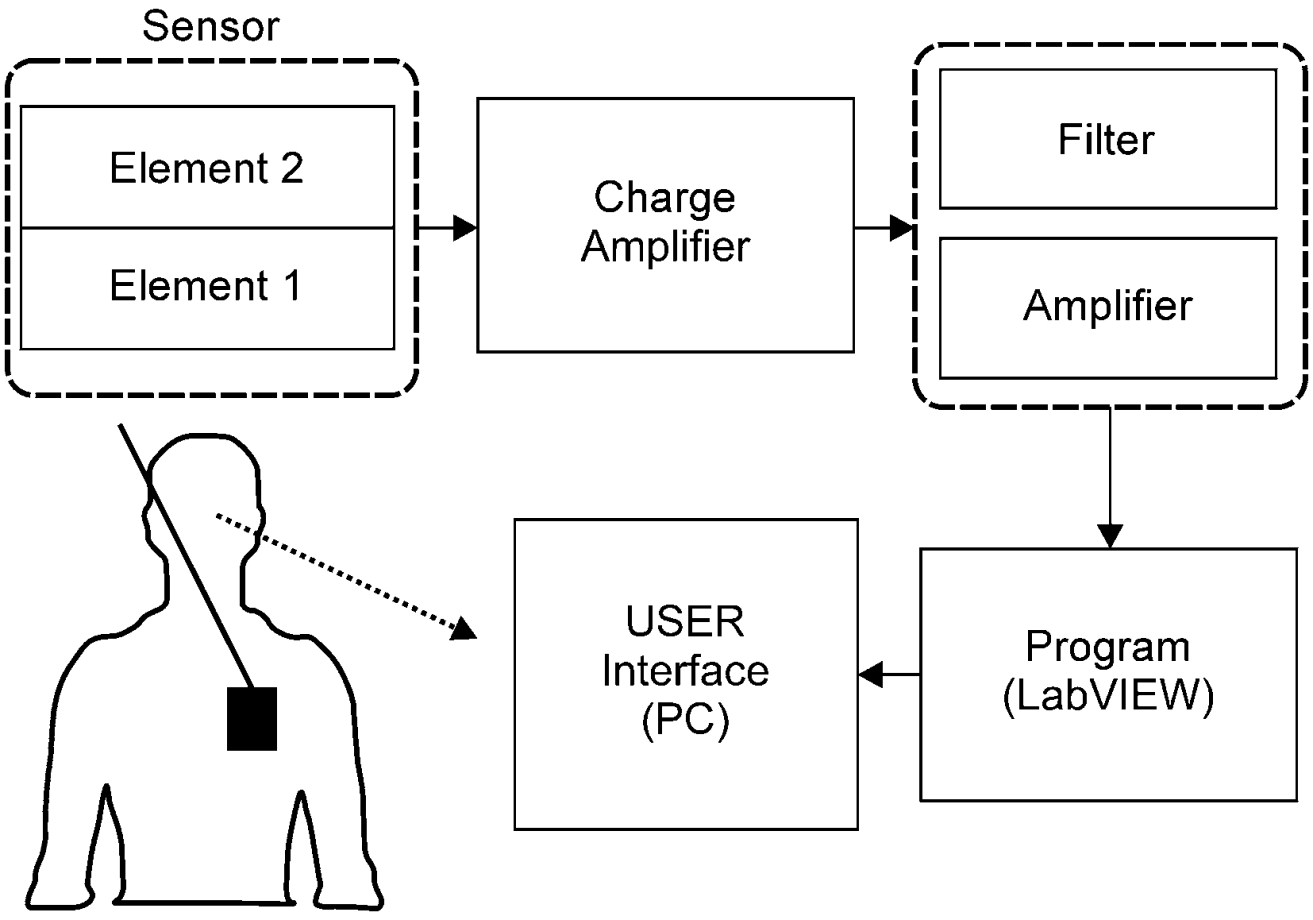
System configuration of the sensor.

To address this, a stacked element sensor is proposed, wherein each of the PVDF is bonded to two PDMS substrates. One element is used to measure movements related to breathing, and the other is used to measure movements related to heart rate. PVDF and PDMS are flexible, lightweight, biocompatible, and inexpensive. The signal generated by the sensor is processed by filters and amplifiers, as shown in Fig. 1, and is displayed by a monitor.

### Piezoelectric theory

As shown in Supplementary Figure S3, when a force F_i_ acts on the PVDF thin film, it causes piezoelectric displacement of the film, which can be expressed as a determinant, as shown in Eq. (1). Here *d*_*ij*_ is the piezoelectric coefficient and T_ij_ is the vertical stress along the axis.

The thickness of the film is very small compared to the width and length, and the charge moves to different surfaces in the thickness direction of the PVDF thin film.

1 $$\left[\begin{array}{c}{D}_{1}\\ {D}_{2}\\ {D}_{3}\end{array}\right]=\left[\begin{array}{ccc}0& 0& 0\\ 0& 0& 0\\ {d}_{31}& {d}_{32}& {d}_{33}\end{array} \begin{array}{ccc}0& {d}_{15}& 0\\ {d}_{24}& 0& 0\\ 0& 0& 0\end{array}\right]\left[\begin{array}{c}{T}_{11}\\ {T}_{22}\\ {T}_{33}\\ {T}_{23}\\ {T}_{13}\\ {T}_{12}\end{array}\right]$$

In general, *d*_*31*_ is 10 times larger than *d*_*32*_; therefore, it can be ignored. When calculated using *d*_*31*_ and *d*_*33*_, the direction of the force is the same as that of axes 1 and 3.

Based on this assumption, the piezoelectric displacement can be expressed as follows:

2 $${D}_{3}={D}_{33}{T}_{33}+{D}_{31}{T}_{11}$$

Equation (3) is used to calculate the generated charge.

3 $$Q={D}_{3}A=({D}_{33}{T}_{33}+{D}_{31}{T}_{11})A$$

### Sensor structure design

Vital signals can be measured in two ways: using insertion and non-insertion methods. The insertion method involves inserting a device, which can cause some physical discomfort to the subject. In this study, the non-insertion method was selected because it is easy to use and does not affect the vital signals.

Supplementary Figure S4 shows the elements that are used for the measurement, each consisting of PVDF and PDMS, 20 mm wide and 30 mm long, to be attached to the chest. The positional changes caused by heartbeat is approximately 0.2–0.5 mm and the changes that occur with breathing is approximately 4–12 mm^11,12^.

The method aims to measure heart rate and respiration rate simultaneously with a single sensor that is easy to use, ergonomic, and cheap. The PVDF used in the sensor is a material that is widely used in the industry and is suitable for the human body^13–16^, and the silicone elastic coating agent PDMS is a material commonly used in general nano and micro systems. PDMS can be made to acquire various properties by mixing with a curing agent in a variable ratio. In this study, PDMS was used to protect flexible substrates and sensing materials^17–20^.

To optimize the thickness of PVDF to the thickness of PDMS, the sensor was analyzed using the finite element method. The thickness of the PDMS layer was 1000 μm, the sensitivity of the sensor was determined by the thickness of the PVDF layer, and the simulated thicknesses were 30 μm, 60 μm, and 100 μm, which are commercially available. Supplementary Table S1 shows the physical properties of PVDF and PDMS.

In the simulation phase, the edge of the sensor was held under a fixed support condition, and a pressure in the range of 1–10 kPa, similar to the heart rate and respiration rate, was applied to the surface.

As shown in Supplementary Figure S5, the displacement with respect to pressure and the generated voltage are the largest in the case of PVDF with a thickness of 30 μm; therefore, a PVDF with a thickness of 30 μm was used for the experiments.

**Figure 2:**
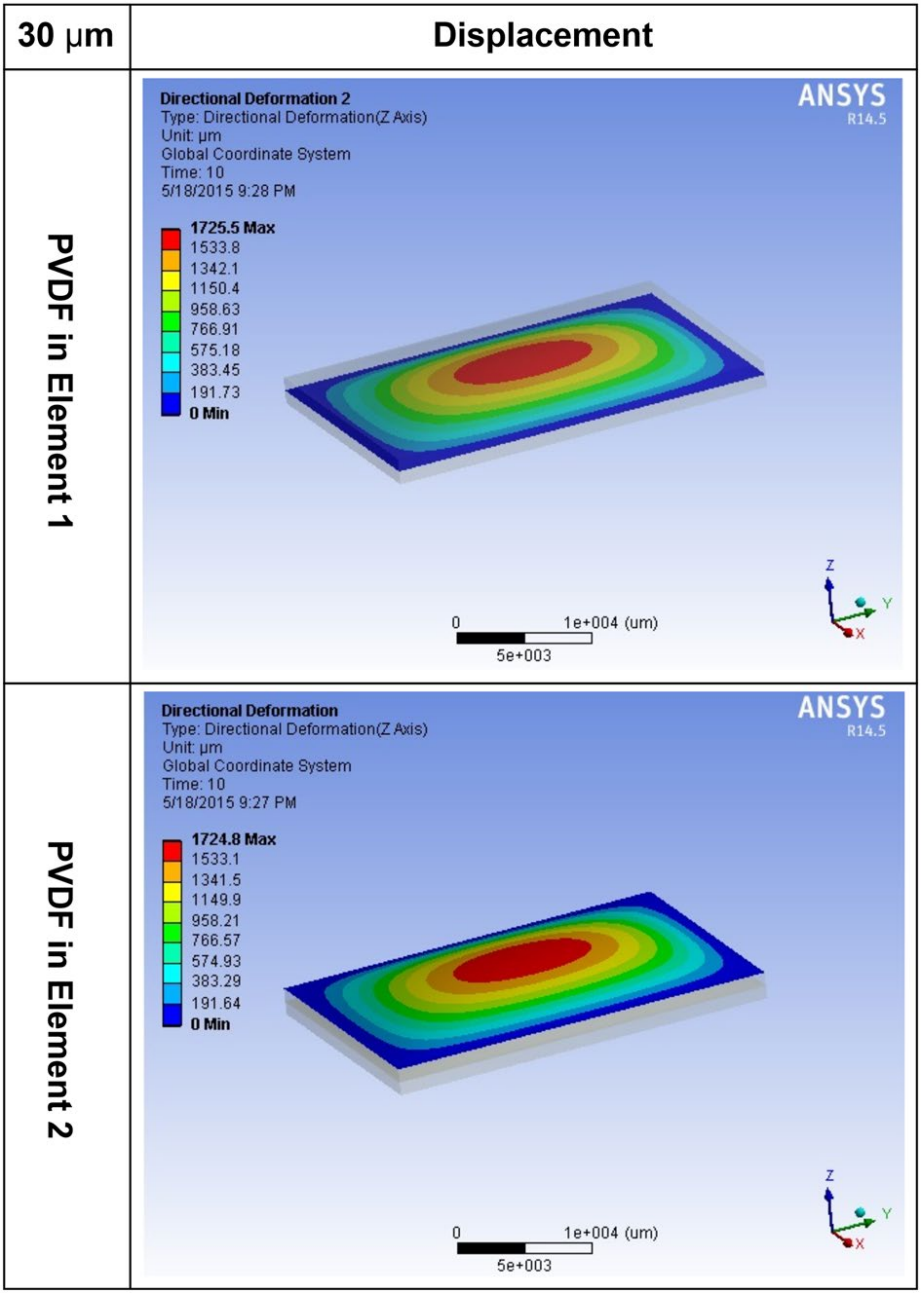
ANSYS result of Elements 1 and 2.

Figure 2 presents the displacement of PVDF with a thickness of 30 μm.

### Signal process design

Several approaches have been reported for the modeling of PVDF materials. Piezopolymers act as capacitive generator-type sensors that provide an electrical output signal without external electrical excitation. At the end of the low-frequency spectrum, a simple R–C model describes the behavior of the electrical device. The piezoelectric sensor operates in the charge and voltage modes, as shown in Supplementary Figure S6. In the charge mode, the signal is transmitted to the interface through the charging integrator. The data on the differential of the pressure is measured directly. Owing to the linear relationship between the pressure-induced deformation of PVDF and the charge due to polarization, the charge mode was adopted in this study.

In the charge mode, if the charge accumulates on both pole plates, the piezoelectric component can be considered a charge source. Thus, the circuit model consists of a charge generator connected in parallel with capacitor Cs and resistor Rs, which is the resistance of the sensor.

#### Charge amplifier design

The advantages of charge amplifiers can be realized by using long cables between the piezo film sensors and electronics. The piezoelectric sensor can be molded, as shown in Supplementary Figure S7. The value of the human pulse rate is small; therefore, the measurements are subjected to errors. Therefore, feedback RC with a large R is necessary. The output voltage depends on the charge generated and capacitor *C*_*f*_ of the feedback loop as follows:

4 $${V}_{out}=\frac{Q}{{C}_{f}}$$

In our circuit, we use a T-network with one capacitor *C*_*f*_ and three resistors *R*_*1*_, *R*_*2*_, and *R*_*3*_. The cutoff frequency of the charged amplifier is

5 $${f}_{cutoff1}=\frac{1}{2\pi {C}_{1}{R}_{1}\left(1+\frac{{R}_{2}}{{R}_{3}}\right)},$$

#### Filter design

The heartbeat and respiration signals are small, and while there is considerable noise, it does not affect the measurements. We used second-order low- and high-pass filters (Supplementary Figure S8) to detect the heartbeat and respiration signals. The second-order filter can give a slope that is twice as high as that of the first-order filter. More importantly, the quality factor Q must be considered. In the first-order filter, the quality factor Q is always equal to 0.707 (<1). In the case of the synthetic circuit, the cutoff frequency was lower than the design value. Therefore, a change in the quality factor was necessary. By adopting the second-order filter, the quality factor Q became larger than 1.

The cutoff frequencies of the second-order low- and high-pass filters are as follows:

6 $${f}_{cutoff2}=\frac{1}{2\pi \sqrt{{C}_{2}{C}_{3}{R}_{4}{R}_{5}}},$$

7 $${f}_{cutoff3}=\frac{1}{2\pi \sqrt{{C}_{4}{C}_{5}{R}_{6}{R}_{7}}}.$$

The frequencies of heartbeat and respiration were 1–2 and 0.2–0.34 Hz, respectively. For heartbeat detection, both low- and high-pass filters must be used. For respiration detection, we need only a low-pass filter with a suitable cutoff frequency.

With components *R*_*1*_ = 200 KΩ, *R*_*2*_ = 1 MΩ, *R*_*3*_ = 1 MΩ, *R*_*4*_ = 1 MΩ, *R*_*5*_ = 10 MΩ, *R*_*6*_ = 330 KΩ, *R*_*7*_ = 680 KΩ and *C*_*1*_ = 1 nF, *C*_*2*_ = 47 nF, *C*_*3*_ = 10 nF, *C*_*4*_ = 0.47 μF, and *C*_*5*_ = 0.47 μF:

- Cutoff frequency of charged amplifier,
  $${f}_{cutoff1}=0.159 \mathrm{Hz}$$
- Cutoff frequency of low- and high-pass filters for heartbeat detection,
  $${f}_{cutoff2}=2.32 \mathrm{Hz}$$
  $${f}_{cutoff3}=0.70 \mathrm{Hz}$$
- Cutoff frequency of the low-pass filter for respiration detection,
  $${f}_{cutoff2}=0.70 \mathrm{Hz}$$

The frequency response of the circuit was simulated using Proteous 7.8 SP2 software, as shown in Supplementary Figure S9. The passband of the heartbeat ranges from 0.7 to 2.32 Hz, and the passband of respiration ranges from 0 to 0.7 Hz. These band passes are suitable for the human heart and respiration rates.

### Ethical approval

Ethical approval was obtained from the Kumoh National Institute of Technology Ethical Review Board. Written informed consent was obtained from all individual participants included in the study. All methods were performed in accordance with the Declaration of Helsinki.

## Fabrication

**Figure 3:**
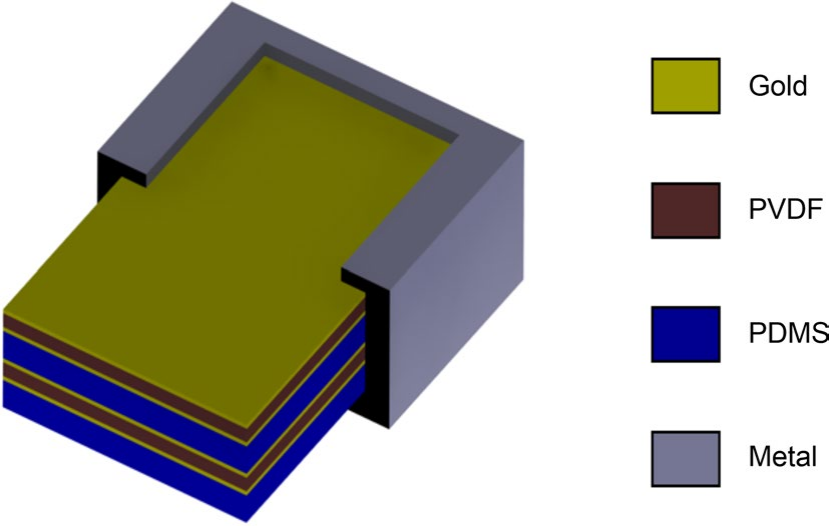
Fabrication process.

The sensor was fabricated in a stack, as shown in Fig. 3.

As the first step, the poled PVDF with a thickness of 30 μm was chosen by optimization; however, it was manufactured without metallization. Then, two electrode layers were deposited on the top and bottom of the PVDF thin film. Gold was favored because of its good conductive properties. Different approaches such as thermal vapor deposition, ion sputtering, and magnetron sputtering were considered. PVDF was deformed by high temperatures during thermal vapor deposition and ion sputtering; therefore, it affected the sensitivity of the thin film. We evaluated magnetron sputtering for gold deposition and patterning using a Cressington 108 sputter coater machine for 3 min.

**Figure 4:**
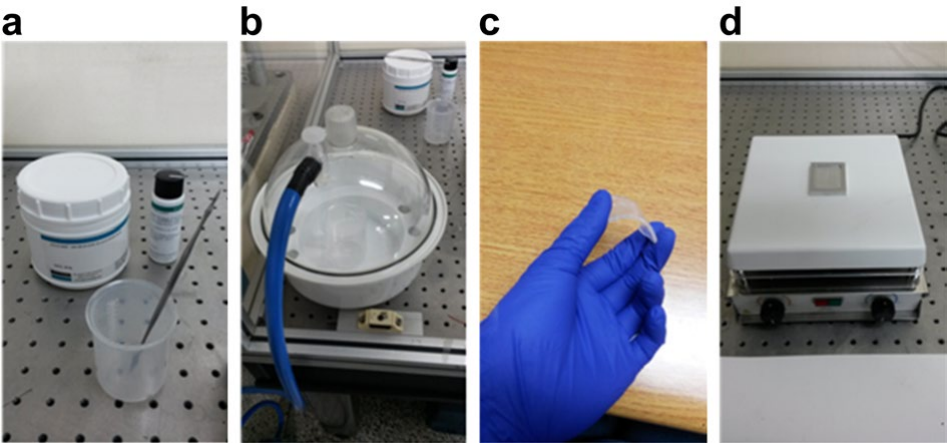
PDMS fabrication process (**a**) Sylgard 184 (**b**) Degassing process (**c**) peeled PDMS (**d**) fabricated PDMS.

In the second step, PDMS was prepared using the mold method. Sylgard 184 (Dow Corning Corporation) elastomer was mixed in a variable ratio by weight to obtain different properties. The sensor must be attached to the human chest, and for this, a 10:1 ratio exhibited good adhesiveness. The PMDS solution was degassed in a vacuum desiccator for 1 h to release the air bubbles. A metal mold was also fabricated. The mold with the PDMS liquid was placed on a hot plate at 100 °C for 10 min. Subsequently, the PDMS layer was peeled from the metal mold (Fig. 4).

The third step is the most crucial step, wherein the PVDF, gold conductive film, and PDMS layer are laminated. Plasma bonding is generally used for lamination, but the process is extremely vulnerable to the bonding of the conductive film and PDMS. Therefore, a thin film with a thickness of approximately 1 µm was coated on the PDMS using a rotation coating method to achieve high attenuation and adhesion.

**Figure 5:**
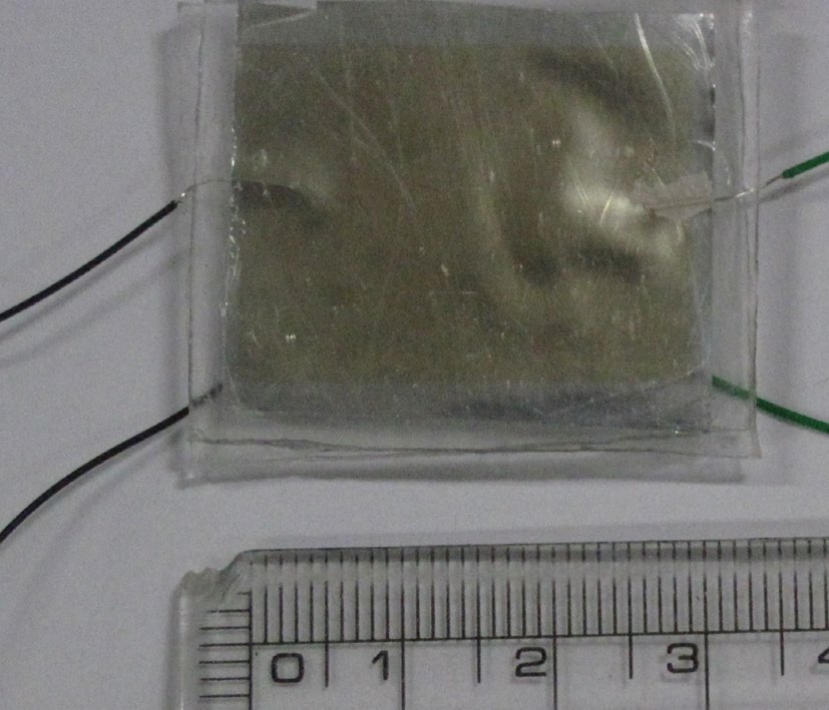
Fabricated sensor.

The last step is to connect the wire that connects the circuit receiving the sensor's output to the gold film with an adhesive. Figure 5 shows the completed stacked sensor.

## Experimental results

### Calibration of sensor

In this experiment, the operation of the sensor was validated by generating an external force and pressure using a stepping motor (Model C7214-9015 Oriential Motor Co. Ltd., Japan). The external force was 0–6 N, and when converted to pressure, it became 0–10 kPa and was measured with a load cell. The load cell was connected to an Adruino Uno R3 board, and the output was converted using a 10-bit ADC.

The output of the sensor corresponding to the force exerted by the stepping motor on elements 1 and 2 were measured, as shown in Supplementary Figure S10. The sensitivity of element 2 was 0.209 V/N and that of element 1 was 0.163 V/N. Supplementary Figure S11 shows the displacement results measured by the displacement sensor (commercial displacement sensor), and Supplementary Figure S12 shows the outputs from the results of Supplementary Figures S10 and S11. According to these experimental results, the sensitivity of the proposed sensor to displacement was 0.002 V/m. Based on this sensitivity, element 1 was set to measure the heart rate and element 2 was set to measure the respiration rate.

### Physical test

**Figure 6:**
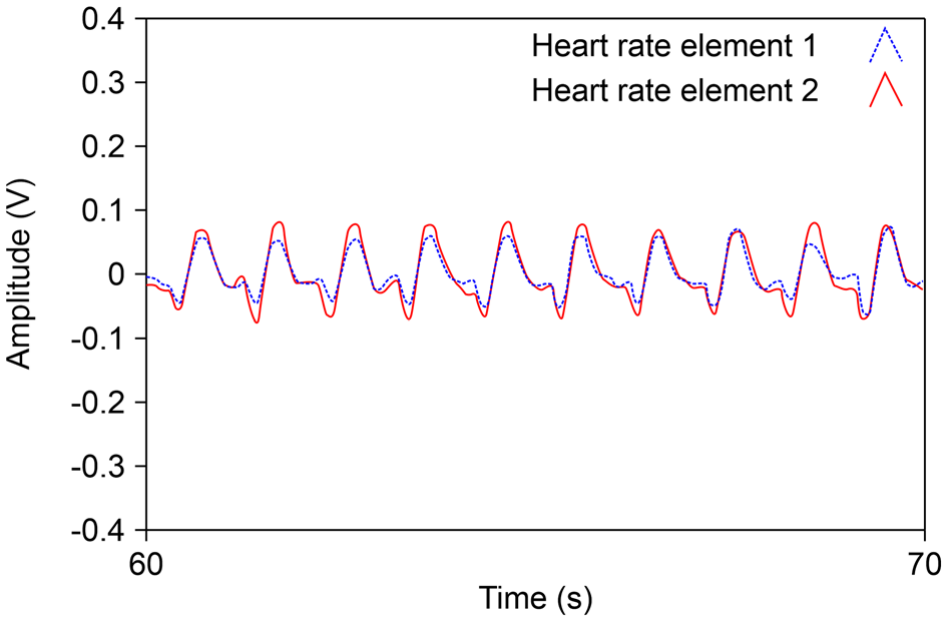
Results of filtering wrist pulse.

The wrist pulse was measured by attaching the first sensor to the human wrist. The signal measured by the sensor was analyzed using LabVIEW. The result of filtering the measured pulse signal is shown in Fig. 6.

Next, it was attached to the chest, and 10 signals were measured. The performance of the sensor proposed in this study was confirmed by comparing the results with those of Citizen CH-456, a digital measuring instrument that measures the heart rate and respiration rate^21^.

**Figure 7:**
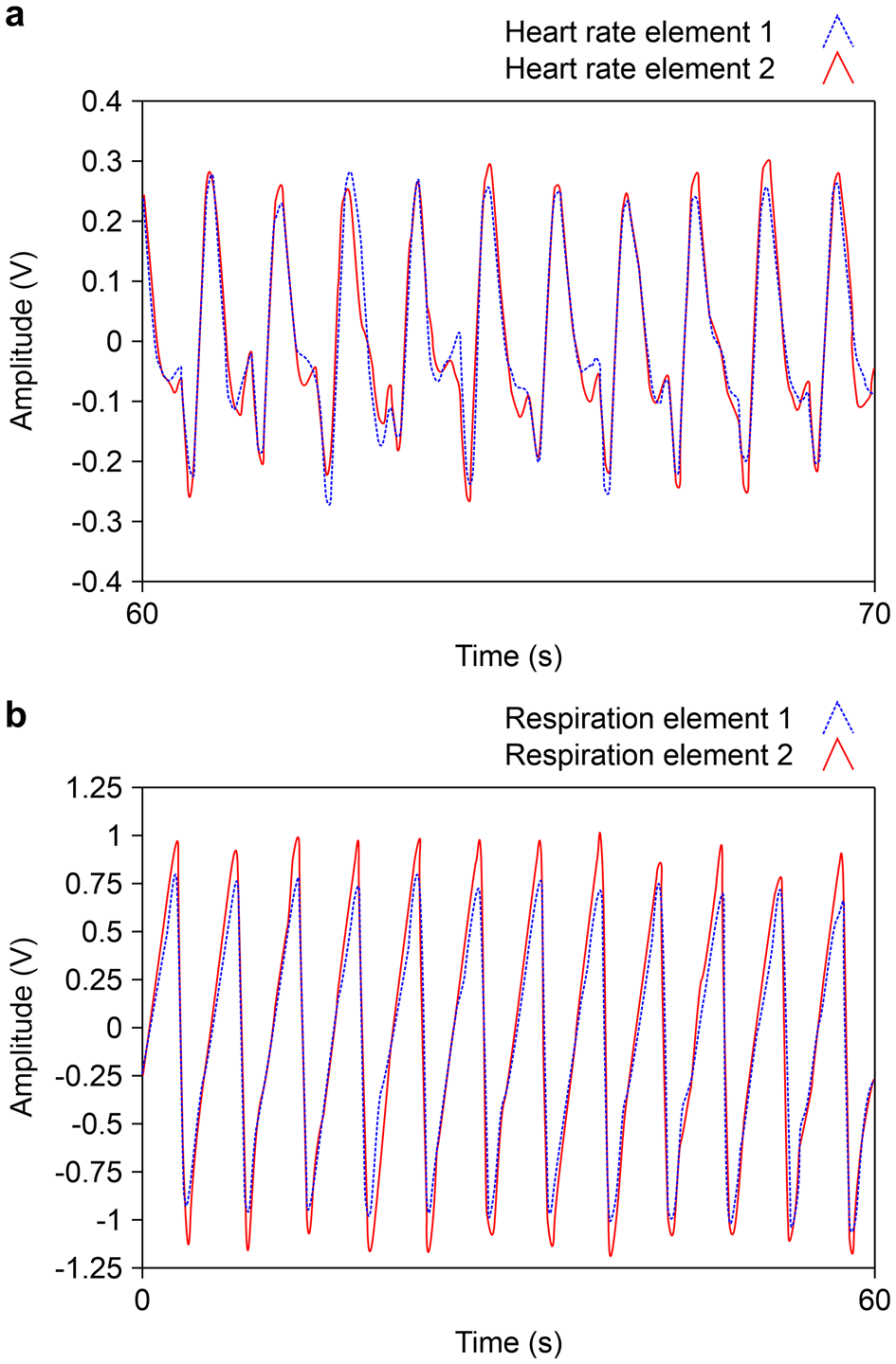
Filtered signal results of heart rate and respiration.

Each element can measure both heart rate and breath, and the external force and pressure generated by breathing are greater than those of the heart rate. To obtain the result, noise was removed, and the signals of heart rate and breath were separated. This process was performed using LabVIEW, and the results are shown in Fig. 7.

In the graph, the blue line represents the signal from element 1, and the red line represents the signal from element 2. Each peak represents one cycle of breathing and heart rate. It can be seen from the graph that the signal from element 2 is greater than that from element 1, which is the same as the result of the mechanical experiment. In addition, it can be confirmed that the same period is observed when the heart rate and pulse signal of the wrist are compared.

**Figure 8:**
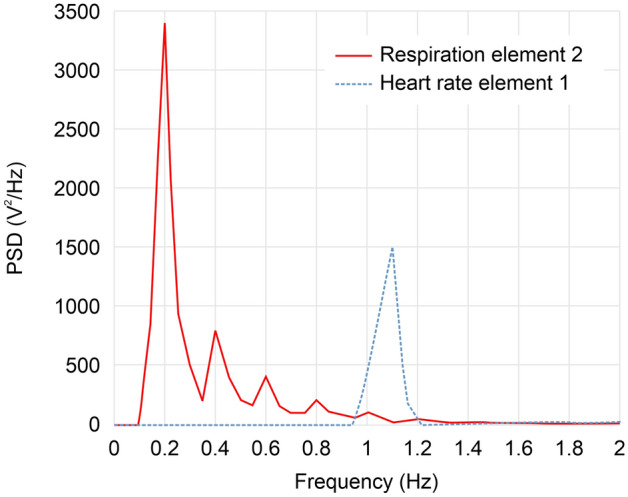
Power spectral density (PSD) of heart rate and respiration signal.

Figure 8 shows the power spectral density (PSD) of the heart rate and breath signal. The peak point of the PSD refers to the frequency of the heart and respiratory rates. In this experiment, the peak value of heart rate was 1.06 Hz, and the peak value of breathing was 0.2 Hz.

Supplementary Table S2 shows the results of the heart rate and respiration rate measured using a commercial sensor and the proposed sensor. This is a measurement of the heart rate and respiration rate per minute, and the sensor's position and muscle movement resulted in a different value from the digital meter's result; however, these errors were insignificant.

## Conclusions

In this study, a stacked sensor using a PVDF piezoelectric film was fabricated to simultaneously measure the heart rate and respiration rate with one sensor. A structure suitable for the human body was designed using PVDF bonded to PDMS, and the sensor was tested with a mechanical method and an actual human body.

The two elements have sensitivities of 0.163 V/N and 0.209 V/N, and in response to force, element 2 had a 28% higher sensitivity characteristic than element 1. In addition, element 2 showed a sensitivity of 0.002 V/D. Therefore, element 2 measured respiration rate and element 1 measured the heart rate. Lastly, for performance verification, a comparison was made with the results of a commercial digital heart rate and respiration rate measuring instrument. Since research on the method of simultaneously measuring human vital signals with multiple sensors is still in its infancy, it needs to be verified through various research pursuits.

## Supplementary Information

Supplementary Information.

## Data Availability

The datasets used and/or analyzed during the current study are available from the corresponding author on reasonable request.
