# Supplementary material for: Simultaneous measurement of two biological signals using a multi-layered polyvinylidene fluoride sensor
Source: Sci Rep. 2022 Jan 27;12:1507. doi: 10.1038/s41598-022-05622-z (PMC8795388; doi:10.1038/s41598-022-05622-z)
Supplement: Supplementary file 1 — Supplementary Information. [file 41598_2022_5622_MOESM1_ESM.docx]

**Supplementary Information**

Supplementary Figures

**Supplementary Figure S1.** Schematic representation of normal ECG


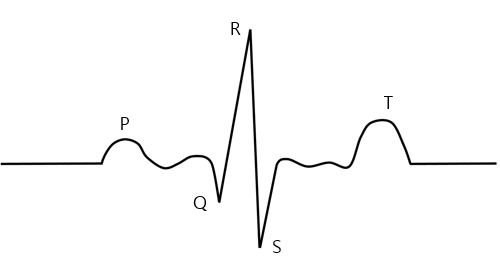


**Supplementary Figure S2.** Heart rate and respiration signals using a sensor with one PVDF thin film


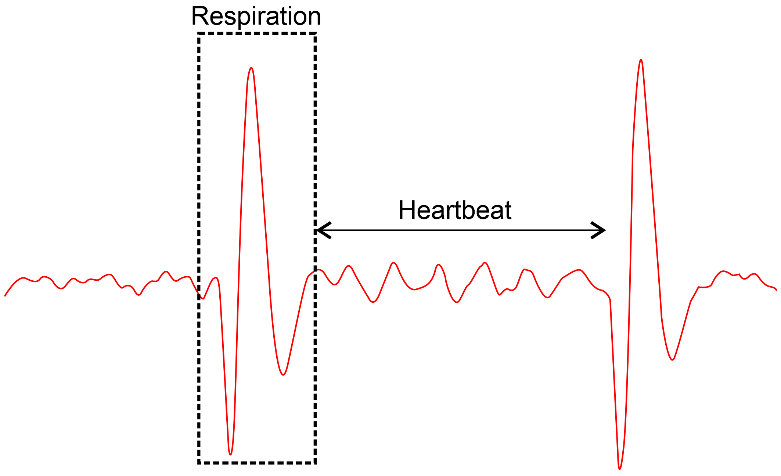


**Supplementary Figure S3. PVDF thin film**


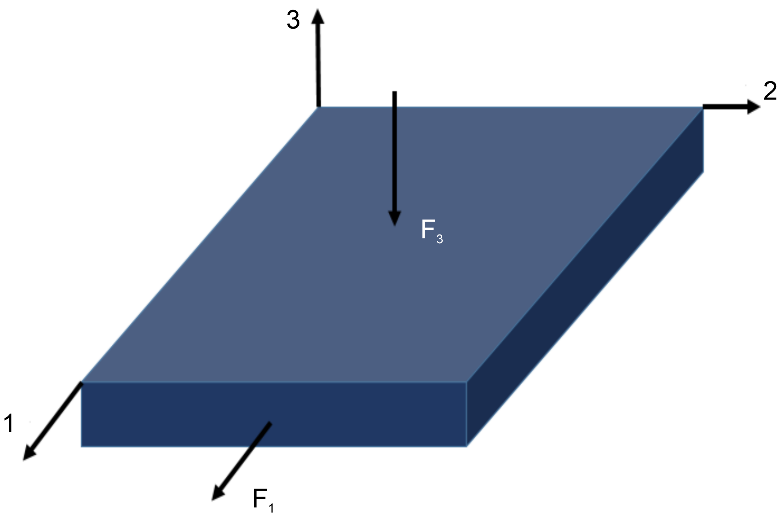


**Supplementary Figure S4.** Stacked element structure


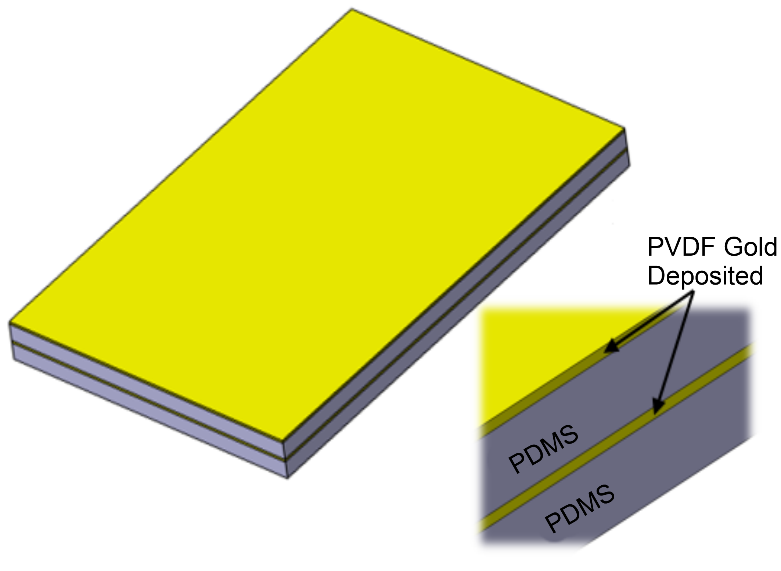


**Supplementary Figure S5.** Displacements of PVDF with sample thickness of 30 μm, 60 μm, and 100 μm


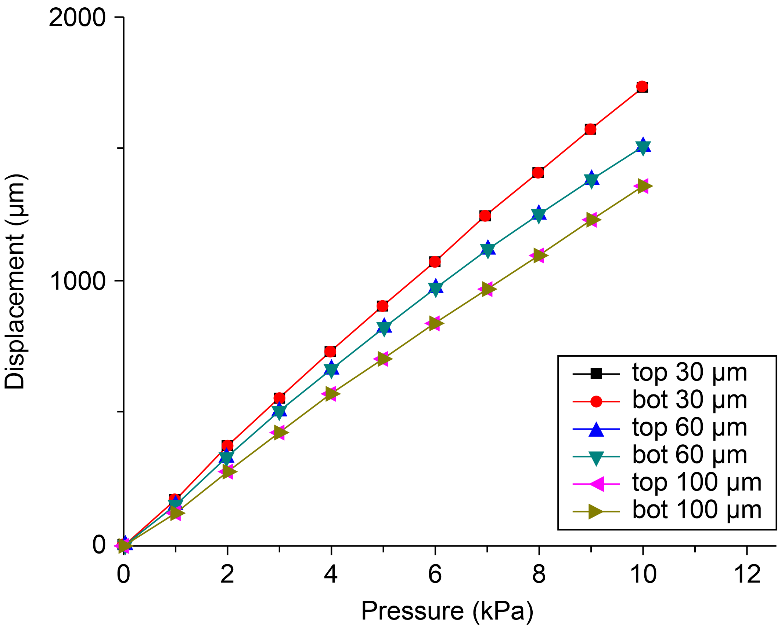


**Supplementary Figure S6.** Voltage and charge modes of a sensor





**Supplementary Figure S7.** Charge amplifier





**Supplementary Figure S8.** Second order (a) low-pass (b) high-pass filter





**Supplementary Figure S9.** Frequency response of heartbeat and respiration signals





**Supplementary Figure S10.** Output voltage of sensor and linearity


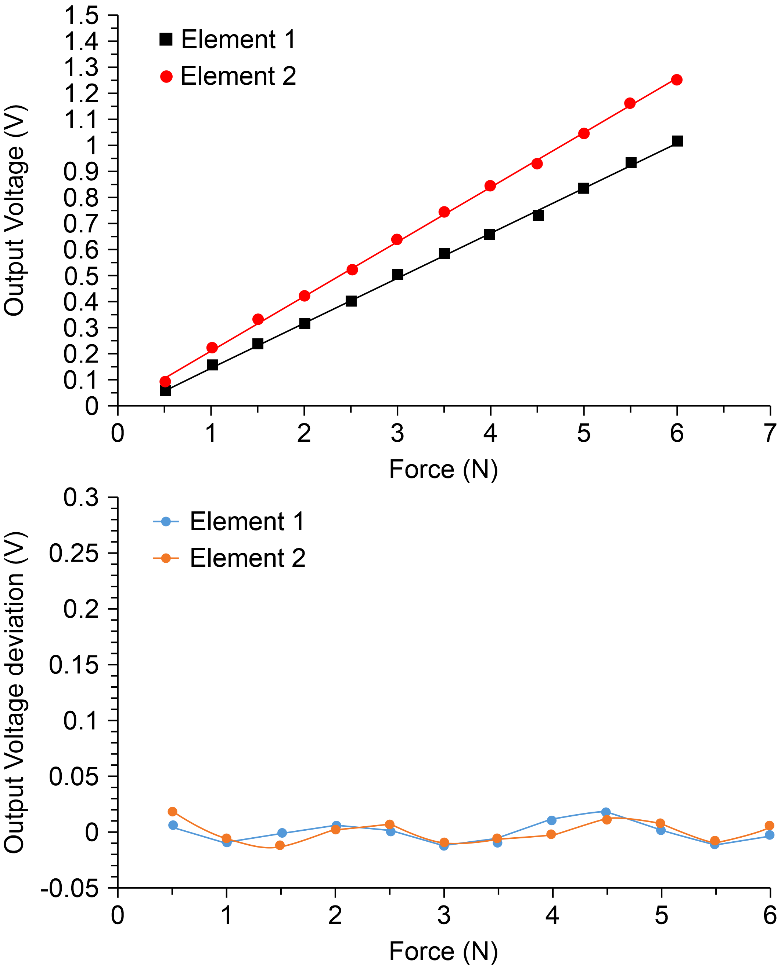


**Supplementary Figure S11.** Force–Displacement results of the sensor


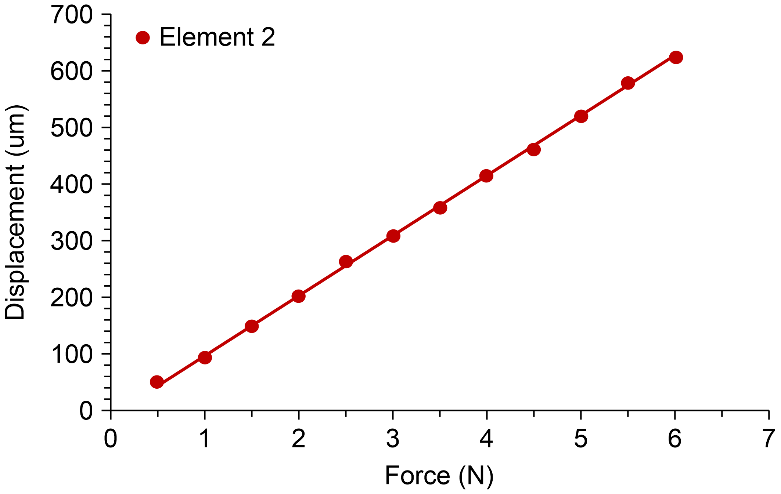


**Supplementary Figure S12.** Displacement–Voltage results of the sensor


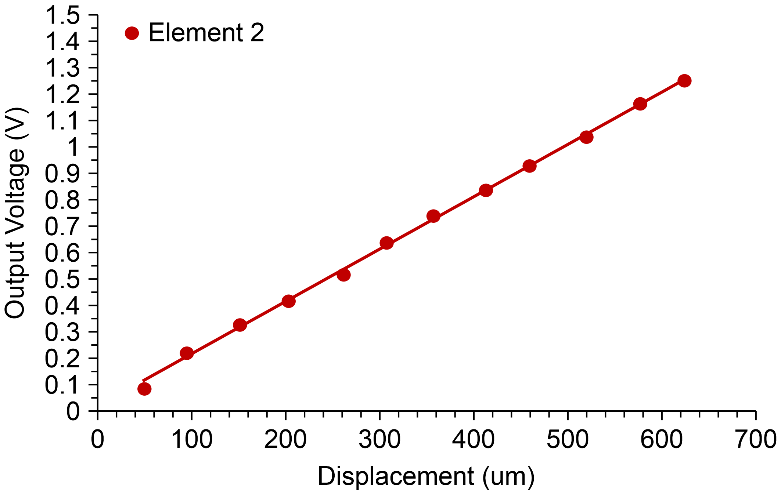


Supplementary Tables

**Supplementary Table S1.** Material properties.

|  | PVDF | PDMS |
| --- | --- | --- |
| Density (kg/m^3^) | 1780 | 965 |
| Young’s modulus (Pa) | 2×10^9^ | 6×10^5^ |
| Poisson’s ratio | 0.3 | 0.4999 |
| Shear modulus | 7.6923×10^8^ | 2.0001×10^5^ |
| *d_31_* | 17 | - |
| *d_32_* | 5 | - |
| *d_33_* | -21 | - |

**Supplementary Table S2.** Comparison of heart rate (HR) and respiration rate (RR) measured by a commercial sensor (c.sensor) and the proposed sensor (p.sensor).

|  | HR  p.sensor | HR  c.sensor | RR  p.sensor | RR  c.sensor |
| --- | --- | --- | --- | --- |
| 1 | 63 times | 65 times | 13 times | 12 times |
| 2 | 62 times | 61 times | 11 times | 12 times |
| 3 | 64 times | 63 times | 12 times | 11 times |
